# An integrated framework for the study of exercise across the postdiagnosis cancer continuum

**DOI:** 10.3389/fonc.2024.1432899

**Published:** 2024-09-23

**Authors:** Kerry S. Courneya, Margaret L. McNeely, Christopher M. Booth, Christine M. Friedenreich

**Affiliations:** ^1^ Faculty of Kinesiology, Sport, and Recreation, College of Health Sciences, University of Alberta, Edmonton, AB, Canada; ^2^ Department of Physical Therapy, Faculty of Rehabilitation Medicine, University of Alberta, Edmonton, AB, Canada; ^3^ Supportive Care Services and Patient Experience, Cancer Care Alberta, Edmonton, AB, Canada; ^4^ Department of Oncology, Queen’s University, Kingston, ON, Canada; ^5^ Cancer Care and Epidemiology, Cancer Research Institute, Queen’s University, Kingston, ON, Canada; ^6^ Department of Cancer Epidemiology and Prevention Research, Alberta Health Services, Calgary, AB, Canada; ^7^ Departments of Oncology and Community Health Sciences, Cumming School of Medicine, University of Calgary, Calgary, AB, Canada

**Keywords:** cancer treatments, combination therapy, exercise, physical activity, multimodality therapy, prehabilitation, rehabilitation

## Abstract

Exercise plays many important roles across the entire cancer continuum that have been described in previous frameworks. These frameworks, however, have generally provided a simplified description of the roles of exercise postdiagnosis. The modern cancer treatment landscape has become complex and often consists of multiple lines of multimodal treatments combined concurrently and/or sequentially and delivered over many months or years. This complexity requires a more multifaceted and targeted approach to the study of exercise after a cancer diagnosis. Here, we propose a new integrated framework—Exercise Across the Postdiagnosis Cancer Continuum (EPiCC)—that highlights the distinct roles of exercise for disease treatment and supportive care from diagnosis until death. We also propose new terminology to clarify the distinct roles of exercise that emerge in the context of the modern cancer treatment landscape. The EPiCC Framework is structured around multiple sequential cancer treatments that highlight six distinct cancer treatment-related time periods for exercise—before treatments, during treatments, between treatments, immediately after successful treatments, during longer term survivorship after successful treatments, and during end of life after unsuccessful treatments. The EPiCC Framework proposes that the specific roles of exercise as a disease treatment and supportive care intervention will vary depending on its positioning within different cancer treatment combinations. As a cancer treatment, exercise may serve as a “priming therapy”, primary therapy, neoadjuvant therapy, induction therapy, “bridging therapy”, adjuvant therapy, consolidation therapy, maintenance therapy, and/or salvage therapy. As a supportive care intervention, exercise may serve as prehabilitation, intrahabilitation, interhabilitation, rehabilitation, “perihabilitation”, health promotion/disease prevention, and/or palliation. To date, exercise has been studied during all of the cancer treatment-related time periods but only in relation to some cancer treatments and combinations. Moreover, fewer studies have examined exercise across multiple cancer treatment-related time periods within any cancer treatment combination. Future research is needed to study exercise as a disease treatment and supportive care intervention within and across the distinct cancer treatment-related time periods contained within different cancer treatment combinations. The aim of the EPiCC Framework is to stimulate a more targeted, integrated, and clinically-informed approach to the study of exercise after a cancer diagnosis.

## Introduction

Exercise plays many important roles across the entire cancer continuum. In 2001, the Physical Exercise Across the Cancer Experience (PEACE) Framework was proposed to organize these roles, bring structure to the field, and highlight new opportunities for exercise oncology research (1). On the postdiagnosis side, the PEACE Framework focused on the supportive care role of exercise across a simple clinical scenario of a single nonsurgical treatment that resulted in either cure or persistent disease. Based on this simple clinical scenario, the PEACE Framework proposed that postdiagnosis exercise may play a supportive care role before treatment (buffering), during treatment (coping), immediately after successful treatment (rehabilitation), immediately after failed treatment (palliation), and during long-term survivorship for patients who were cured (health promotion and survival).

The PEACE Framework was updated in 2007 as the Physical Activity and Cancer Control (PACC) Framework (2) to incorporate new ideas regarding the cancer continuum and to highlight the potential role of exercise in affecting clinical cancer outcomes proposed in the Organizational Framework of Physical Activity and Clinical Endpoints in Cancer Survivors (3). On the postdiagnosis side, PACC was still based on the simple clinical scenario of a single nonsurgical treatment with exercise proposed to play a potential role before treatment (treatment preparation/coping), during treatment (treatment effectiveness/coping), immediately after treatment (recovery/rehabilitation), during long-term survivorship (disease prevention/health promotion), and at the end of life (palliation and survival). Although the PEACE and PACC Frameworks acknowledged the complexity of cancer treatments, this complexity was not explicitly incorporated into the frameworks. Consequently, both frameworks provided a relatively simple description of the potential roles of exercise after a cancer diagnosis.

More recently, the Multiphasic Prehabilitation Framework was proposed to describe the potential roles of multimodal prehabilitation (including exercise) for both patients and caregivers across the postdiagnosis cancer continuum (4). This framework highlighted the potential roles of multimodal prehabilitation across multiple consecutive cancer treatments from diagnosis until treatment completion. Although this framework described the potential role of exercise across a more complicated treatment landscape, it did not explicitly address the posttreatment phase of the cancer continuum or the advanced/metastatic disease setting. Moreover, the framework primarily described opportunities for prehabilitation during the treatment phase, and did not address the potential role of exercise as a disease treatment.

In 2022, the Exercise as Cancer Treatment (EXACT) Framework was proposed to provide a more detailed assessment of the potential role of exercise as a cancer treatment from a clinical oncology perspective (5, 6). The EXACT framework proposed that exercise may have effects on the primary tumor, micrometastases, and metastatic disease before, during, and/or after other cancer treatments (5, 6). Although the EXACT Framework provided a more clinically focused assessment of exercise as a cancer treatment and highlighted the issue of treatment sequencing, it did not fully incorporate the complexity of multiple cancer treatments and it did not address the supportive care role of exercise.

Here, we propose a new integrated framework—Exercise Across the Postdiagnosis Cancer Continuum (EPiCC)—that highlights the dual roles of postdiagnosis exercise for supportive care and disease treatment across the modern cancer treatment landscape from diagnosis until death. The primary goal of proposing this new framework is to better characterize existing exercise oncology research and to identify new opportunities for future research that emerge in the context of the modern cancer treatment landscape. A secondary goal is to propose terminology for describing the complicated supportive care and disease treatment roles of exercise across the postdiagnosis cancer continuum to foster consistency and specificity across studies.

## The modern cancer treatment landscape

The modern cancer treatment landscape has become complex with the addition of newer treatments such as immunotherapies and targeted therapies to previously established treatments such as surgery, radiation therapy, chemotherapy, and endocrine (hormone) therapy. With the rapid expansion of treatment options, the treatment landscape for many cancers has become saturated and complicated. As a result, the optimal combination and sequencing of cancer treatments has become a critical issue in clinical oncology with implications for treatment efficacy as well as safety and tolerability (7–9). Modern cancer treatment approaches often consist of multiple lines of multimodal treatments combined concurrently and/or sequentially to treat a disease that can progress or recur multiple times. Cancer patients may receive many different treatments over many months or years. The terminology used to describe this increasingly complex cancer treatment landscape is not fully standardized and is sometimes inconsistent (10). **Table 1** provides definitions for some of the key terms used to describe cancer treatment combinations and sequencing that are important to understand when evaluating the role of exercise as a cancer treatment in the modern treatment landscape.

**Table 1:** Terminology and definitions for describing cancer treatment combinations and sequencing^1^.

Terminology	Definition/Description
Treatment modality	Generally refers to a broad type or method of treatment. The most common treatment modalities in cancer are surgery, radiation therapy, chemotherapy, hormone therapy, targeted therapy, and immunotherapy. Modalities may be further categorized as local therapies that treat a specific organ or area of the body (e.g., surgery, radiation therapy, some drug therapies) or systemic therapies (drugs) that treat the entire body (e.g., chemotherapy, hormone therapy, targeted therapy, and immunotherapy). Exercise may be considered a systemic therapy and classified as its own cancer treatment modality.
Treatment scheduling and sequencing	Treatment scheduling refers to the administration plan for a single treatment modality (e.g., chemotherapy, immunotherapy) including the specific type (e.g., specific drugs or type of radiation therapy), administration method (e.g., by mouth, injection, infusion, external beam), dose (e.g., milligrams, grays), frequency (e.g., once per day, every 3 weeks), and length of treatment (e.g., 6 weeks, 6 months). The treatment schedule for exercise is the exercise prescription including the type, frequency, intensity, duration, progression, periodization, and length of program. Treatment sequencing refers to the administration plan for one treatment in relation to another treatment.
Monotherapy	Generally refers to the use of a single treatment modality to treat cancer (e.g., surgery, radiation therapy) or the use of a single drug (agent) to treat cancer (e.g., endocrine monotherapy, pembrolizumab monotherapy). Exercise is essentially a single drug (energy expenditure) and, therefore, may be considered a monotherapy when administered by itself (i.e., single agent exercise or exercise monotherapy).
Combination therapy	Generally refers to more than one method of therapy. Combination therapy may refer to more than one treatment modality (i.e., multimodal therapy) or more than one drug within the same systemic modality (e.g., combination chemotherapy or combination immunotherapy). Combination therapy may be given concurrently (at the same time) and/or sequentially (one after the other). A single drug administered on its own may still be referred to as monotherapy even if it is part of a sequential combination (e.g., surgery followed by endocrine monotherapy). Similarly, exercise may be referred to as a monotherapy when administered on its own in a sequential combination with other cancer treatments (before, between, or after) but referred to as a concurrent therapy when administered at the same time as other cancer treatments (during).
First-line therapy	Generally refers to the first treatment(s) that provide the best opportunity for cure, remission, or benefit (i.e., Plan A). First-line therapy may consist of a single modality, especially in the advanced stage setting, but often includes a combination of multimodal treatments given concurrently and/or sequentially in the early stage setting (e.g., surgery followed by chemotherapy followed by radiation therapy).
Primary therapy	Generally refers to the most definitive treatment(s) for a cancer regardless of its sequencing with the goal of cure or remission. Usually reserved for surgery or radiation therapy in the early stage setting, however, it may also be used to describe a systemic therapy (e.g., primary chemotherapy) in the advanced stage setting.
Neoadjuvant therapy	Generally refers to upfront nonsurgical treatment(s) for nonmetastatic disease with the goal of reducing the size/extent of the primary tumor and eliminating micrometastases prior to definitive surgery. Usually reserved for nonsurgical therapies prior to definitive surgery.
Induction therapy	Generally refers to upfront nonsurgical treatment(s) prior to other nonsurgical treatments for a hematological (blood) cancer or advanced solid cancer with the goal of remission or cure. Usually reserved for chemotherapy or other systemic therapies that will be followed by radiation therapy or other systemic therapies.
Adjuvant therapy	Generally refers to nonsurgical treatment(s) after primary surgery for nonmetastatic disease with the goal of killing any remaining cancer cells. Usually reserved for radiation therapy or systemic therapies after definitive surgery.
Consolidation therapy	Generally refers to nonsurgical treatment(s) after induction therapy for a hematological (blood) cancer or advanced solid cancer with the goal of killing any remaining cancer cells after the induction treatment. Usually reserved for systemic therapies after radiation therapy or after other systemic therapies.
Maintenance therapy	Generally refers to nonsurgical treatment(s) for any stage cancer given at a lower dose for an extended period of time with the goal of maintaining cure, remission, or stable disease. Usually reserved for systemic therapies after effective primary/induction and adjuvant/consolidation treatments.
Salvage therapy	Generally refers to second-line (or later line) treatments for any stage cancer that has not responded or has stopped responding to the first-line treatments (i.e., Plan B, Plan C, etc.). Most often reserved for radiation therapy or systemic therapies but may include surgery.
Priming therapy	Generally refers to an upfront nonsurgical treatment(s) prior to other nonsurgical treatments with the goal of priming or preparing the cancer cells to be more sensitive to the subsequent treatment. The priming therapy usually has minimal anticancer effects itself but may alter the cancer cells or tumor microenvironment in a way that makes them more vulnerable to the subsequent therapies.
Bridging therapy	Specifically refers to treatment given between apheresis (collection of T-cells) and the infusion of chimeric antigen receptor (CAR) T-cell therapy. The goal of bridging therapy is to prevent disease progression while waiting for the preparation of the CAR T-cell therapy. More broadly, bridging therapy may refer to treatment given between any two treatments where the patient is waiting for the subsequent treatment because of medical or logistical issues.

^1^Adapted from the National Cancer Institute’s Dictionary of Cancer Terms (https://www.cancer.gov/publications/dictionaries/cancer-terms) accessed February 5, 2024.

Integrating exercise into this convoluted cancer treatment landscape has itself become more complicated. As a result, it is no longer adequate to describe the role of postdiagnosis exercise in reference to a single nonsurgical cancer treatment (i.e., before, during, or after) or in reference to an isolated cancer treatment-related time period (e.g., exercise during chemotherapy, postsurgical exercise). Exercise must be situated within the entirety of the broader cancer treatment landscape. Moreover, it is insufficiently precise to discuss exercise as a “cancer treatment” or to refer to its supportive care roles as “rehabilitation” and “prehabilitation”. The complexity of the modern cancer treatment landscape requires a more multifaceted and sophisticated approach to the study of exercise after a cancer diagnosis. To articulate the role of exercise in the modern cancer treatment landscape, it is important to first characterize the properties of exercise as a cancer treatment.

## Exercise as a cancer treatment

As noted in the EXACT Framework (5), the potential roles of exercise as a cancer treatment are determined by its unique characteristics. Exercise is essentially a single drug (energy expenditure) whose biological effects can be manipulated based on the type, dose, frequency, intensity, and duration (i.e., the exercise prescription). Like other drugs, exercise has systemic effects which means it can potentially act as a treatment for local, regional, and distant disease. Although a single administration of exercise (i.e., an exercise session) can have acute biological effects (11), it will not instantly eliminate an existing tumor like a surgical resection or ablative therapy. In this sense, exercise is like other nonsurgical therapies such as radiation therapy or chemotherapy that require multiple administrations over an extended period of time to achieve a clinical benefit. In terms of cancer treatment terminology (**Table 1**), exercise may be considered a systemic therapy that is its own treatment modality (i.e., exercise therapy).

## Integrating exercise into the modern cancer treatment landscape

From an exercise perspective, there are two important features of current cancer treatments that influence how exercise may be integrated into the modern cancer treatment landscape. With respect to cancer treatment modalities, the important distinction is between treatment modalities that involve a single administration of the therapy with immediate therapeutic effects (usually surgery or ablative therapies) versus treatment modalities that involve multiple administrations of a therapy with gradual or continuous therapeutic effects over time (usually radiation therapy and systemic therapies). Exercise generally cannot be performed during the actual administration of any cancer therapy (except perhaps during a continuous infusion), however, it can be performed within (between) multiple administrations of the same cancer therapy while the treatment is having therapeutic effects (i.e., during treatment). Conceptually, exercise during treatment refers to exercising while the treatment is working or having therapeutic effects. Pragmatically, however, exercise during treatment may refer to exercise between the first and last administrations of a therapy that is administered on multiple occasions over time. The critical point is that exercise can be performed before, during, and after therapies that require multiple administrations over time (usually nonsurgical therapies) whereas it can only be performed before and after therapies that consist of a single administration (usually surgery or ablative therapies).

With respect to cancer treatment combinations, the important distinction is between concurrent (at the same time) and sequential (one after the other) combinations. Exercise can be performed between sequential combinations that have a sufficient break between them (e.g., 4-6 weeks) but not between concurrent combinations or sequential treatments with minimal breaks between them. Consequently, exercise may be performed before, during, between, and after sequential combinations whereas it can only be performed before, during, and after concurrent combinations. These two important distinctions influence the potential roles of exercise across the modern cancer treatment landscape. **Table 2** provides suggested terminology and definitions regarding the timing of exercise in relation to the modern cancer treatment landscape.

**Table 2:** Proposed terminology and definitions for describing the timing of exercise across the postdiagnosis cancer continuum.

Exercise Timing Terminology	Exercise Timing Definition
Exercise Postdiagnosis	Exercise after a cancer diagnosis without regard to cancer treatments.
Exercise Pretreatment	Exercise after a cancer diagnosis and before the first administration of any cancer treatment.
Exercise Within Treatment (during infusion therapy)	Exercise during the actual administration of a cancer treatment. Exercise within treatment may only be possible during a continuous infusion and, therefore, may be better described as “exercise during infusion therapy”.
Exercise During Treatment	Exercise between the first and last administrations of a treatment that is delivered in multiple administrations over an extended period of time (e.g., radiation therapy, chemotherapy, immunotherapy, chemoradiation therapy).
Exercise Between Treatments	Exercise during any break between two planned sequential cancer treatments (or any break within the same treatment).
Exercise During a Line of Therapy	Exercise between the first and last administrations of multiple sequential treatments in a planned line of therapy (includes exercise during and between multiple treatments).
Exercise Posttreatment	Exercise shortly after the last treatment administration in a line of therapy when no further treatments are planned.
Exercise During Survivorship	Exercise after short term recovery from curative treatment(s) when no further treatments are planned.
Exercise Between Lines of Therapy	Exercise during any break between two distinct lines of therapy when multiple lines of therapy are likely or expected.
Exercise During End of Life	Exercise after the last administration of an unsuccessful treatment for progressive disease when no further treatments are planned.
Exercise Across the Postdiagnosis Cancer Continuum	Exercise from the time of diagnosis and for the balance of life.

## Common modern cancer treatment paradigms

There are endless treatment combinations for different cancers and disease stages that make it virtually impossible to describe the roles of exercise succinctly across all clinical scenarios. Nevertheless, there are some general treatment paradigms that can help exercise oncology researchers think through the role of exercise after a cancer diagnosis. **Table 3** presents six common cancer treatment paradigms (and their terminology) that are typical for different disease stages in solid tumors and hematologic cancers. The major distinction among the modern cancer treatment paradigms from an exercise perspective is the occurrence and timing of surgery.

**Table 3:** Common cancer treatment paradigms and terminology by disease stage at diagnosis.

Treatment Paradigm	Disease Stage	Cancer Treatment Paradigm^1^			Setting
		Treatment A	Treatment B	Treatment C	
Deferred treatment	≤I	If Necessary	If Necessary	If Necessary	Active surveillance
Upfront surgery	I/II	Surgery (Primary)	Nonsurgical^2^ (Adjuvant)	± Systemic (Maintenance)	Adjuvant
Delayed surgery	II/II	Nonsurgical^2^ (Neoadjuvant)	Surgery (Primary)	± Nonsurgical^2^ (Adjuvant)	Neoadjuvant
No surgery (curative)	I-III	Radiation Therapy (Primary)	± Systemic (Adjuvant)	± Systemic (Maintenance)	Curative nonsurgical
		Systemic (Induction)	Radiation Therapy (Primary)	± Systemic (adjuvant)	
		Systemic (Induction)	Systemic (Consolidation)	± Systemic (Maintenance)	
No surgery (treatable)	III-IV	Nonsurgical^2^ (First-line/Primary)	± Systemic (Second-line/Salvage)	± Systemic (Third-line/Salvage)	Advanced/Metastatic
No treatment (progressive)	IV	None	None	None	Palliative

^1^Treatments A, B, and C may include multiple concurrent and/or sequential nonsurgical therapies. ^2^Nonsurgical is radiation therapy or systemic therapy.

For many early stage solid tumors (stages I and II), surgery occurs first (upfront) followed by possible nonsurgical therapies (i.e., the adjuvant setting). For some locally advanced solid tumors (stages II and III), surgery may occur after nonsurgical therapies (i.e., the neoadjuvant setting) and be followed by additional nonsurgical therapies. For hematological cancers and some early stage solid tumors, curative treatment may not include surgery at all (i.e., curative settings without surgery). Similarly, surgery is uncommon in the metastatic setting (stage IV) where the treatment sequence typically involves multiple lines of systemic therapies, although in some circumstances local therapies may be used to treat limited metastases (i.e., “metastasis-directed therapy”) (12). Finally, there are two scenarios in which treatment may not occur at all. For some very small low grade cancers (stage ≤I), treatment may only occur if necessary (i.e., the active surveillance setting) whereas for some widely disseminated cancers there may be no effective treatments available (i.e., the palliative setting).

## Exercise across the postdiagnosis cancer continuum framework

**Figure 1:**
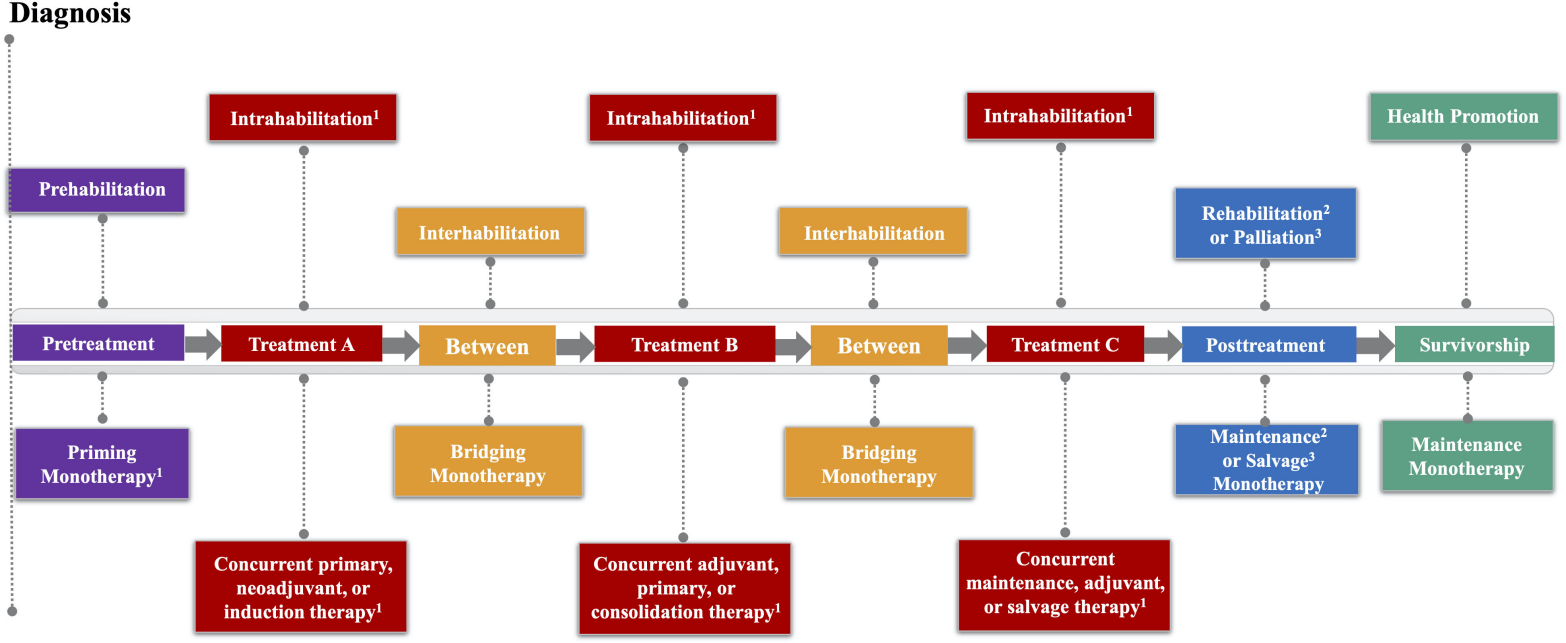
Exercise Across the Postdiagnosis Cancer Continuum (EPiCC) Framework. ^1^If treatment is not surgery; ^2^If cure/remission; ^3^If progressive disease.

The EPiCC Framework integrates and expands upon previous frameworks that have described the roles of exercise across the postdiagnosis cancer continuum (1–6). More specifically, the EPiCC Framework proposes two broad roles for exercise—supportive care and disease treatment—across the postdiagnosis cancer care continuum from diagnosis until death (**Figure 1**). The supportive care role of exercise generally focuses on quality of life issues and includes outcomes such as health-related fitness, physical functioning, symptom/side effect management, treatment tolerance, and psychosocial functioning. The disease treatment role of exercise generally focuses on quantity of life issues and includes outcomes related to tumor control, disease control, and survival. Although the modern cancer treatment landscape is diverse and complex, the EPiCC Framework portrays three sequential treatments as sufficient to capture the diverse roles exercise might play across multiple sequential cancer treatments. The EPiCC Framework highlights six distinct cancer treatment-related time periods for exercise—before treatments, during treatments, between treatments, immediately after successful treatments, during survivorship after successful treatments, and during end of life after unsuccessful treatments. The primary proposition of the EPiCC Framework is that the effects of exercise on disease and supportive care outcomes will vary depending on its positioning within different cancer treatment combinations. For example, even across a clinical oncology scenario of three sequential nonsurgical cancer treatments as depicted in the EPiCC Framework, there are 255 possible exercise combinations (including all 1-way to 8-way combinations from pretreatment to survivorship).

**Figure 2:**
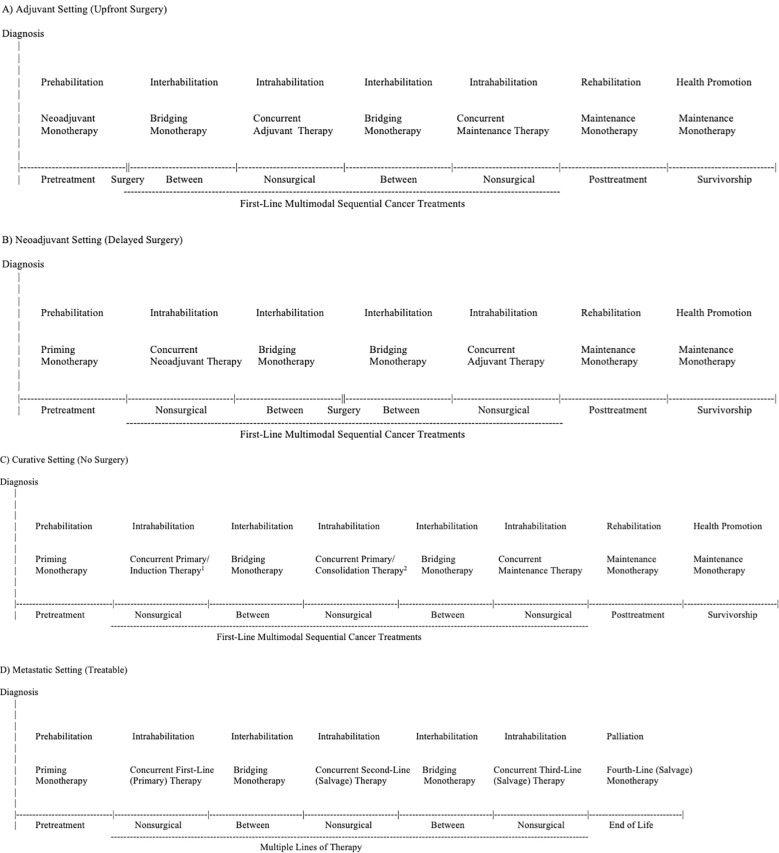
Exercise Across the Postdiagnosis Cancer Continuum (EPiCC) Framework Tailored to the Adjuvant (Upfront Surgery) Setting **(A)**, Neoadjuvant (Delayed Surgery) Setting **(B)**, Curative (No Surgery) Setting **(C)**, and Metastatic (Treatable) Setting **(D)**. ^1^Primary therapy if radiation therapy; induction therapy if systemic therapy. ^2^Primary therapy if radiation therapy; consolidation therapy if systemic therapy.

As a cancer treatment, exercise may serve as a “priming therapy”, primary therapy, neoadjuvant therapy, induction therapy, “bridging therapy”, adjuvant therapy, consolidation therapy, maintenance therapy, and/or salvage therapy depending on its positioning within different cancer treatment combinations (**Table 1**). As a supportive care intervention, exercise may serve as prehabilitation, intrahabilitation, interhabilitation, rehabilitation, “perihabilitation”, health promotion/disease prevention, and/or palliation depending on its timing in relation to cancer treatments (**Table 4**). **Figure 2** provides adaptations of the general EPiCC framework to the four main cancer treatment paradigms that involve multiple sequential cancer treatments: (a) the adjuvant setting (upfront surgery), (b) the neoadjuvant setting (delayed surgery), (c) the nonsurgical setting (curative), and (d) the metastatic setting (treatable). The following sections provide a general description and conceptual overview of exercise as a disease treatment and supportive care intervention in the EPiCC Framework.

**Table 4:** Proposed terminology and definitions for describing the supportive care roles of exercise across the postdiagnosis cancer continuum.

Supportive Care Terminology	Supportive Care Description/Definition
Prehabilitation Exercise	Exercise after a cancer diagnosis and before the first cancer treatment to help patients improve functioning and better tolerate and recover from the impending treatment(s).
Intrahabilitation Exercise	Exercise during a nonsurgical cancer treatment to help patients maintain functioning and better manage (tolerate) the current treatment. Depending on when the nonsurgical treatment occurs in a treatment sequence, intrahabilitation may also include rehabilitation from any previous cancer treatment(s) and/or prehabilitation for any subsequent cancer treatment(s).
Interhabilitation Exercise	Exercise during a break between two sequential cancer treatments (or lines of therapy) to help patients recover from previous treatment(s) and/or prepare for subsequent treatment(s). Interhabilitation exercise may include both rehabilitation and prehabilitation goals.
Rehabilitation Exercise	Exercise after successful completion of curative cancer treatment(s) to help patients restore functioning and better recover from the treatment(s).
Perihabilitation Exercise	Exercise around a specific cancer treatment or line of treatments including before, during, between, and/or after to help patients prepare, manage, and/or recover from the treatment(s). Perihabilitation exercise may include prehabilitation, intrahabilitation, and rehabilitation goals.
Health Promotion and Disease Prevention Exercise	Exercise after rehabilitation from successful curative cancer treatments to help patients improve their overall health and prevent secondary diseases/late effects during longer-term survivorship.
Palliative Exercise	Exercise after unsuccessful cancer treatments for progressive disease to help patients palliate disease symptoms and lingering treatment side effects.

## Exercise as treatment within the modern cancer treatment landscape

The expanding cancer treatment landscape offers new opportunities for exercise to serve as a cancer treatment by demonstrating independent, additive, and/or synergistic effects with existing cancer treatments (13). The role of exercise as a cancer treatment may vary across the postdiagnosis cancer continuum depending on its combination and sequencing with other cancer treatments. Exercise mechanisms may inform the optimal positioning of exercise in relation to different cancer treatments (i.e., exercise sequencing). In general, exercise may cause biological and/or hemodynamic effects in the tumor microenvironment and systemically that independently, or in combination with other cancer treatments, affect the growth and spread of cancer cells (6). It is unlikely, however, that exercise alone is curative for any cancer. Preclinical studies have shown that exercise alone, at best, slows the progression of cancer (14–17). No preclinical studies have demonstrated that exercise alone produces complete responses, disease regression (partial responses), or even stable disease. Consequently, for clinical benefit, exercise will need to be combined with other cancer treatments either concurrently or sequentially.

In the following sections, we briefly describe the potential roles of exercise as a cancer treatment based on its positioning within different treatment combinations. We use the term “concurrent exercise therapy” to refer to exercise that is administered at the same time as another cancer treatment (i.e., during that treatment) whereas we use the term “exercise monotherapy” to refer to exercise that is administered asynchronously (i.e., by itself) even though it is part of a sequential treatment combination (i.e., before, between, or after other treatments).

### Exercise as primary therapy

Primary therapy generally refers to the most definitive treatment(s) for a cancer with the goal of cure or remission and is usually reserved for surgery or radiation therapy in the early stage setting (**Table 1**). In the advanced/metastatic setting, primary therapy may be used to describe a systemic therapy. Exercise monotherapy is unlikely to be considered a primary therapy for any cancer because it does not eliminate or even dramatically reduce cancerous tumors in preclinical models (14–17). Moreover, most cancers have at least one effective treatment that makes it unlikely exercise would even be examined as a primary monotherapy. The only clinical scenario that provides an opportunity to examine exercise as a primary monotherapy is the active surveillance setting in which no immediate treatments are provided. Similar to preclinical models, however, exercise as a primary monotherapy may slow the progression of cancer but it is unlikely to eliminate or reverse the disease (18, 19).

It is possible that exercise could serve as a concurrent primary therapy with radiation therapy (or a systemic therapy) based on preclinical studies suggesting that exercise may enhance radiosensitivity and drug sensitivity/delivery through improvements in tumor vasculature, perfusion, and hypoxia (20). Conceptually, this treatment role for exercise is similar to neoadjuvant and induction therapy because exercise is combined concurrently with the first upfront nonsurgical treatment. It is different, however, because a primary therapy is expected to be the definitive treatment for the disease whereas neoadjuvant and induction therapies are considered preliminary therapies to be followed by definitive surgery or nonsurgical therapies, respectively.

Exercise monotherapy prior to a nonsurgical primary therapy (or neoadjuvant or induction therapy) may be effective because alterations in the tumor microenvironment may enhance the effectiveness of subsequent nonsurgical therapies such as radiation therapy, chemotherapy, and immunotherapy. This possible treatment role for exercise may be better described as a “priming monotherapy” (21) rather than a primary (or neoadjuvant or induction monotherapy) because exercise may alter the cancer or tumor microenvironment to be more responsive to the upfront nonsurgical therapy but it will not induce a remission or cure itself. This treatment role for exercise has not been studied in preclinical models because exercise is usually not administered between the time of an established primary tumor and an upfront nonsurgical cancer treatment. It has also not been studied in clinical research because exercise interventions have generally not occurred before an upfront nonsurgical therapy. It is unclear, therefore, if there is sufficient time between diagnosis and upfront nonsurgical therapies (whether primary, neoadjuvant, or induction) for exercise interventions to be feasible and effective as a priming monotherapy.

### Exercise as neoadjuvant therapy

Neoadjuvant therapy generally refers to upfront nonsurgical therapies given for nonmetastatic disease with the goal of reducing the size/extent of the primary tumor and micrometastases prior to definitive surgery (**Table 1**). Exercise is unlikely to serve as a neoadjuvant monotherapy because it fails to produce any tumor shrinkage on its own in preclinical models (14–17). Moreover, exercise effects on the tumor microenvironment may not be clinically helpful in the neoadjuvant setting because the tumor is usually completely resected after the neoadjuvant therapy. It is possible that improved tumor vasculature may reduce the number of tumor cells being shed into the surrounding tissue and blood vessels before surgery, however, the window for such an effect may be short. Nevertheless, studies of exercise monotherapy prior to surgery may provide important mechanistic evidence on the biological effects of exercise on human *in vivo* tumors (22, 23). As noted earlier, exercise as a “priming monotherapy” prior to a neoadjuvant therapy may enhance the efficacy of the neoadjuvant therapy through tumor vasculature mechanisms, however, the feasibility and efficacy of such a treatment role for exercise has not been tested in preclinical or clinical models.

Exercise as a concurrent neoadjuvant therapy may be clinically efficacious because alterations in the tumor microenvironment may enhance the effectiveness of the concurrent neoadjuvant therapies such as radiation therapy, chemotherapy, and immunotherapy. These effects have been demonstrated in preclinical and clinical studies (24). In preclinical studies, exercise has improved the delivery and efficacy of some systemic therapies by improving tumor vasculature and perfusion (25). In small exploratory clinical studies, exercise has improved the response to neoadjuvant chemoradiation and chemotherapy (24).

For example, Morielli et al. (26) examined the effects of exercise in 36 rectal cancer patients receiving neoadjuvant chemoradiotherapy prior to definitive surgery and reported that the number of patients achieving a pathologic complete or near complete response was significantly (*p*=0.020) higher in the exercise group (10/18 = 56%) compared to the usual care group (3/17 = 18%). Similarly, in a non-randomized trial involving 39 esophageal cancer patients during neoadjuvant chemotherapy (27), the exercise group compared to nonexercise group achieved higher rates of tumor regression (75% *vs*. 37%; p=0.025) and combined tumor and node downstaging (43% *vs*. 16%; p=0.089). Most recently, Sanft et al. (28) examined the effects of a combined diet and exercise intervention on chemotherapy tolerance and pathologic complete response in 173 breast cancer patients initiating chemotherapy. In a subgroup analysis of the 72 women who received neoadjuvant chemotherapy, the intervention group was significantly (p=0.037) more likely to achieve a pathologic complete response (53%) compared to the usual care group (28%). Based on these preliminary data, a definitive phase III trial is examining the effects of exercise as a concurrent neoadjuvant therapy on pathologic complete response in 790 breast cancer patients receiving neoadjuvant chemotherapy (29).

### Exercise as induction therapy

Induction therapy generally refers to upfront systemic treatment(s) in a sequence of systemic treatments for a hematological (blood) cancer or advanced solid cancer with the goal of remission or cure (**Table 1**). Exercise is unlikely to serve as an induction monotherapy because it will not produce a remission or cure on its own (14–17). As noted earlier, exercise monotherapy prior to an induction therapy may be effective because alterations in multiple tumor microenvironments (typical of hematologic cancers or advanced solid tumors) may enhance the effectiveness of subsequent induction therapies such as chemotherapy and immunotherapy. Again, this possible treatment role for exercise may be better described as a “priming monotherapy” rather than an induction monotherapy because it may alter the tumor microenvironment to be more responsive to an induction therapy but it will not induce a remission or cure itself. As noted earlier, this treatment role for exercise has not been studied in preclinical or clinical models. Exercise may be more feasible and helpful as a concurrent induction therapy because alterations in multiple tumor microenvironments (20) may also enhance concurrent therapies, similar to its potential role as a concurrent primary or neoadjuvant therapy (although primary and neoadjuvant therapy usually target a single primary tumor).

### Exercise as adjuvant therapy

Adjuvant therapy generally refers to nonsurgical therapies after primary surgery (or after primary radiation therapy) for nonmetastatic disease with the goal of killing any remaining cancer cells (**Table 1**). Exercise may serve as an adjuvant monotherapy because of the limited number of disseminated cancer cells that typically remain after definitive surgery (30). Exercise as a monotherapy may act as a treatment for treatment naïve micrometastases through various systemic mechanisms including increased fluid shear stress, enhanced immune surveillance, reduced inflammation, and improved insulin sensitivity (31). Exercise has been studied as an adjuvant monotherapy in preclinical animal models that have injected a small number of cancer cells that disseminate in the absence of a primary tumor (32). These studies have generally shown that exercise alone can reduce the number and size of metastatic tumors (i.e., slow disease progression) but not eliminate the remaining cancer cells (32).

There are some clinical scenarios where exercise could be tested as an adjuvant monotherapy although many early stage disease settings with complete surgical resection are followed by some adjuvant therapy. Nevertheless, some observational studies have demonstrated associations between exercise and cancer recurrence or death in clinical scenarios that involved only surgery or limited adjuvant therapy. For example, Friedenreich et al. (33) examined the associations between postdiagnosis physical activity and survival in 425 endometrial cancer patients who had received surgery but only about one-third received any adjuvant therapy. Higher postdiagnosis recreational physical activity was strongly associated with improved disease-free survival (HR, 0.33; 95% CI, 0.17 to 0.64) suggesting that exercise may be active as an adjuvant monotherapy in this clinical setting. Similarly, Lee et al. (34) examined the associations between physical activity and cancer outcomes in 43,596 colorectal cancer survivors from the Korean National Health Insurance Service database. In a subgroup analysis of survivors who had received surgery only, higher physical activity was associated with a significantly lower risk of mortality (HR=0.75; 95% CI, 0.65 to 0.87) suggesting that exercise may serve as an adjuvant monotherapy in this clinical setting.

Exercise that occurs between the first therapy (e.g., primary or neoadjuvant or induction) and second therapy (e.g., adjuvant or primary or consolidation), or between any two therapies, may be considered a “bridging monotherapy” (**Table 1**). The goal of a bridging therapy is to contain or control the cancer while waiting for the next major therapy to be administered. This treatment role for exercise has not been studied in preclinical models because exercise is usually not administered between two sequential cancer treatments. There is a modest amount of clinical research that has examined exercise between neoadjuvant therapy and primary (surgical) therapy which has yielded promising results (26, 35). The relative utility of exercise as a bridging monotherapy, however, may depend on the length of time between two sequential therapies.

Exercise may also be helpful as a concurrent adjuvant therapy. As noted earlier, exercise may directly treat micrometastases through various mechanisms (31), however, it may also potentiate the effects of existing cancer treatments through similar mechanisms (e.g., increased fluid shear stress may make cancer cells more vulnerable to chemotherapy (36), or increased immune surveillance may enhance the effectiveness of immunotherapies (37). Exercise might also improve tumor vasculature and blood flow to small micrometastatic formations depending on their location and, therefore, enhance delivery of systemic therapies to these undetected lesions. Some preclinical studies have examined this scenario by using metastasis models treated with chemotherapy and shown reduced tumor growth and spread with exercise (24). In one clinical study (38), 242 early stage breast cancer patients receiving chemotherapy were randomized to usual care (n=82), aerobic exercise (n=78) or resistance exercise (n=82). In an exploratory follow-up (39), disease-free survival 8 years later was 82.7% for the two exercise groups combined compared with 75.6% for the usual care group (Hazard ratio =0.68, 95% CI=0.37-1.24; log-rank p=0.21) suggesting that exercise may be beneficial as a concurrent adjuvant therapy in this clinical setting.

### Exercise as consolidation therapy

Consolidation therapy generally refers to systemic treatment(s) after upfront (induction) systemic treatment(s) for a hematological (blood) cancer or metastatic disease with the goal of killing any remaining cancer cells after successful induction treatment. It is possible that exercise may serve as a consolidation monotherapy if there are few remaining cancer cells throughout the body after the induction therapy, similar to its role as an adjuvant therapy. Nevertheless, the induction therapy may alter the genetics, biology, tumor microenvironment, and location of any remaining cancer cells making them more or less sensitive to exercise (40). Exercise may also be effective as a concurrent consolidation therapy based on mechanisms previously discussed for concurrent adjuvant therapy. To date, exercise has not been studied as a consolidation therapy (concurrent or monotherapy) in any preclinical or human studies.

### Exercise as maintenance therapy

Maintenance therapy generally refers to systemic treatment(s) after successful primary/adjuvant or induction/consolidation therapies for any stage cancer given at a lower dose for an extended period of time with the goal of maintaining cure, remission, or stable disease (**Table 1**). Exercise may be an effective maintenance monotherapy in the curative setting because of the very limited disease that typically remains after primary and adjuvant treatments. There are possible mechanisms for exercise as a maintenance monotherapy similar to adjuvant monotherapy (31), however, as a maintenance therapy exercise needs to be able to eliminate previously treated micrometastases which may have acquired new mutations or reside in difficult to reach locations (41). Exercise has not been shown to serve as a maintenance monotherapy in preclinical models because studies have not examined exercise after successful previous treatments.

Most current observational studies have tested exercise as a maintenance monotherapy because postdiagnosis assessments of physical activity have generally occurred well after treatments have been completed (e.g., between 2 to 5 years postdiagnosis) (6). Friedenreich et al. (42) summarized 136 studies on PA and cancer outcomes across various cancer types. Results showed that higher postdiagnosis physical activity was significantly associated with a lower risk of cancer-specific mortality (HR=0.66; 95% CI=0.59-0.73), suggesting that exercise may serve as a maintenance monotherapy across a wide range of early stage clinical settings and different treatment combinations.

The Colon Health and Life-Long Exercise Change (CHALLENGE) trial is examining exercise as a maintenance monotherapy in colon cancer survivors (43). CHALLENGE will examine the effects of a 3-year structured exercise program on disease-free survival in high risk stage II or stage III colon cancer patients who have completed surgical resection and adjuvant chemotherapy within the past 2-6 months. CHALLENGE will answer the question of whether exercise as a maintenance monotherapy can lower the risk of recurrence and death in colon cancer patients after surgical resection and adjuvant chemotherapy.

Exercise may also serve as a maintenance monotherapy in the active surveillance setting where newly diagnosed cancer patients do not immediately receive any treatments. Unlike other settings, the maintenance monotherapy would be for treatment naïve disease. Moreover, any cancer treatment in this setting would need to be highly tolerable because treatment toxicities would not be acceptable given the very low risk of cancer death in these patients. Nevertheless, many of these patients will experience cancer progression and will ultimately require cancer treatments (44). For these patients, exercise may also serve as a “priming monotherapy” because it may alter the tumor microenvironment to make it more susceptible to future nonsurgical treatments (radiation therapy, hormone therapy). Given that it is unknown ahead of time which patients might progress to definitive treatment, exercise for cancer treatment during active surveillance may serve as both a maintenance monotherapy and priming monotherapy.

Exercise may be helpful as a concurrent maintenance therapy based on biological mechanisms and mechanical sheer stress (31) which may make micrometastases more vulnerable to other maintenance therapies such as such as chemotherapy, immunotherapy, or hormone therapy, similar to the concurrent adjuvant therapy setting. Some observational studies in breast cancer may have addressed this treatment role because exercise was likely assessed during long-term endocrine therapy, however, most studies have not explicitly analyzed exercise as a maintenance monotherapy versus concurrent therapy (6).

### Exercise as salvage therapy

Salvage therapy generally refers to second-line (or later-line) nonsurgical treatments for any stage cancer that has recurred, not responded, or stopped responding to the previous treatment (**Table 1**). Exercise is unlikely to serve as a salvage monotherapy given that patients have stopped responding to treatments and have extensive disease that has likely acquired many resistance mechanisms. Exercise as a concurrent salvage therapy may be more likely to be effective given its possible role in remodeling tumor microenvironments and facilitating drug delivery to existing tumors (20). No previous preclinical studies and very few observational or intervention studies have tested exercise as a salvage therapy.

Some human studies have examined the effects of exercise during treatment for metastatic disease but few have reported whether the treatments were first-line (primary) or later-line (salvage) therapies. In a systematic review and meta-analysis of 11 studies of exercise and survival outcomes in patients with advanced cancer, exercise was associated with improved survival in 7 observational studies but not in 4 randomized controlled trials (45). The Healthy Exercise for Lymphoma Patients (HELP) trial (46) randomized 122 lymphoma patients to usual care or 12 weeks of supervised aerobic exercise. At the time of the exercise intervention, 54 lymphoma patients were receiving chemotherapy, about half as a first-line therapy (primary therapy) and half as a later-line therapy (salvage therapy). An exploratory analysis revealed that the exercise group had a non-statistically significant (p=0.24) higher clinical complete response to chemotherapy (13/28 = 46.4%) compared to the usual care group (8/26 = 30.8%) suggesting the possibility that exercise may serve as a concurrent salvage therapy (or primary therapy) with chemotherapy in patients with lymphoma cancer.

## Exercise as supportive care within the modern cancer treatment landscape

The expanded cancer treatment landscape also provides many additional opportunities to study exercise as a supportive care intervention. Multiple sequential cancer treatments may make the supportive care benefits of exercise even more important. Exercise may produce even larger and more clinically relevant benefits for health-related fitness, physical functioning, psychological functioning, quality of life, symptom/side effects, treatment tolerance, and possibly even treatment delays (within and between multiple sequential treatments or lines of therapy). In the EPiCC Framework, exercise may play distinct supportive care roles before treatments, during treatments, between treatments, immediately after successful treatments, during long term survivorship after successful treatments, and during end of life after unsuccessful treatments.

Currently, the supportive care terminology for exercise oncology has generally been limited to “prehabilitation” and “rehabilitation”, which were originally used to describe exercise interventions before and after surgery, respectively (4). While these terms can easily be extended to describe exercise interventions before and after nonsurgical cancer treatments (4) (e.g., exercise prehabilitation for chemotherapy or exercise rehabilitation after radiation therapy), they do not adequately describe exercise interventions during or between cancer treatments, during survivorship, or during end of life. Some researchers have proposed that terms such as rehabilitation (47–49), prehabilitation (4, 49), palliative care (50), or health promotion (51) can be expanded to include most or all of the postdiagnosis cancer continuum, however, these expanded definitions may mask the distinct supportive care roles of exercise across the postdiagnosis cancer continuum. Although there may be some overlap of supportive care goals across the postdiagnosis cancer continuum, the EPiCC Framework proposes that each cancer treatment-related time period has a distinct primary supportive care goal directly related to the treatment period.

Conceptually, the goals of prehabilitation exercise are to improve function before an invasive cancer treatment to help buffer or reduce impending treatment complications and side effects. Conversely, the goals of rehabilitation exercise are to recover or restore function after an invasive cancer treatment to help ameliorate any resulting treatment complications and side effects. Seemingly, the goals of exercise during an invasive cancer treatment would be to prevent decline or maintain function to help manage or mitigate emerging treatment complications and side effects. Preventing decline during a cancer treatment is distinct from prehabilitation and rehabilitation.

The concept of “habilitation” typically refers to helping persons with disabilities keep, learn, or improve skills and functioning for daily living (52). Another broader definition of habilitation, however, is the act or process of becoming fit or of making fit for a particular purpose (Dictionary.com). This latter definition fits the general goals of exercise for supportive care in cancer. Moreover, habilitation is the root word of prehabilitation and rehabilitation and can easily be modified to accommodate exercise during or within treatment (intrahabilitation), between treatments (interhabilitation), and even around treatments (perihabilitation) (53).

In the EPiCC Framework, supportive care terminology for exercise is based on the cancer treatment-related time periods. That is, exercise may be performed before treatments (prehabilitation), during treatments (intrahabilitation), between treatments (interhabilitation), immediately after successful treatments (rehabilitation), around treatments (perihabilitation), during longer term survivorship after successful treatments (health promotion/disease prevention), and during end of life after unsuccessful treatments (palliation). **Table 4** provides suggested terminology and definitions for the various supportive care roles of exercise across the postdiagnosis cancer continuum. We now discuss these concepts and the distinct supportive care roles of exercise in more detail.

### Prehabilitation and rehabilitation

As noted previously, the most common terms used to describe exercise after a cancer diagnosis have been prehabilitation and rehabilitation (4). There is no consensus on the definition and scope of prehabilitation (54–56), however, the classic definition restricts it to the period after diagnosis and before the first cancer treatment (57). Others restrict it to the period after diagnosis and before primary surgery which allows exercise during and after neoadjuvant therapy to be considered prehabilitation (58). Still, others include multiple periods from cancer diagnosis until adjuvant therapy which includes before, during, and between multiple sequential cancer treatments (4). Alternatively, given that the term prehabilitation implies preparation for treatments, it could be argued that there must be at least one remaining treatment modality for exercise to be considered prehabilitation in the modern cancer treatment landscape. These different conceptualizations of prehabilitation are depicted in **Table 5**.

**Table 5:** Different conceptualizations of exercise prehabilitation in the context of the modern cancer treatment landscape.

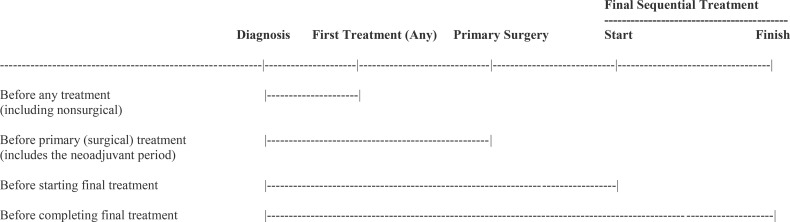

In theory, exercising at any time before a planned cancer treatment may include prehabilitation goals even if it is the second or third cancer treatment in a sequence (**Table 6**). The time between a cancer diagnosis and first cancer treatment is often fairly short (days to weeks) whereas the time between a cancer diagnosis and the second or third cancer treatment in a sequential combination may be substantial (weeks to months). This extended time period makes prehabilitation an even more powerful concept that could play a critical role in cancer care because of the extended sequential cancer treatment protocols (4). The concept of prehabilitation could even be extended to clinical scenarios with multiple lines of therapy where further lines are expected (e.g., bladder, ovarian) or to the active surveillance setting where definitive treatment may be anticipated at some point (59).

**Table 6:** Exercise habilitation concepts according to the Exercise Across the Postdiagnosis Cancer Continuum (EPiCC) framework.

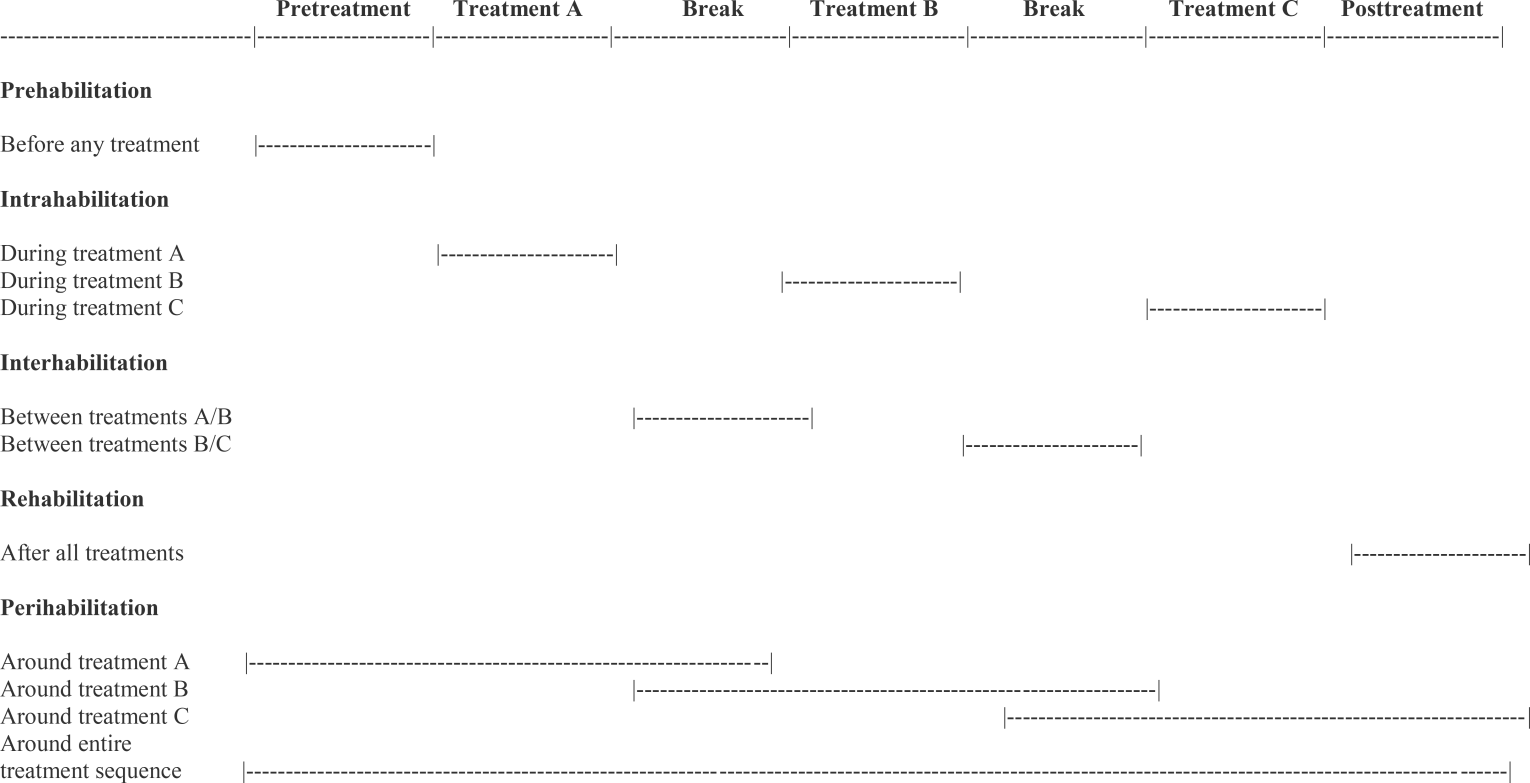

The classic definition of rehabilitation is to restore or recover function after an adverse event (48). Theoretically, rehabilitation may be needed after the very first cancer treatment. Patients can be rehabilitated from a single treatment or from multiple sequential treatments as part of a line of therapy (**Table 6**). Rehabilitation may extend for several months after the final treatment in a multiple sequential treatment paradigm until the patient is fully recovered from treatment effects. Therefore, rehabilitation may start after the first treatment in a series of sequential cancer treatments and extend several months after the final treatment.

In juxtaposing the two concepts, prehabilitation may begin immediately after the cancer diagnosis and extend until just before the last administration of the final sequential therapy (e.g., last chemotherapy infusion, last immunotherapy injection, last radiation therapy fraction). Conversely, rehabilitation may begin immediately after the first administration of the first sequential therapy (e.g., surgery, first chemotherapy infusion, first radiation therapy fraction) and extend beyond the last administration of the final sequential therapy (e.g., last chemotherapy infusion, last immunotherapy injection, last radiation therapy fraction) until full recovery. Consequently, prehabilitation and rehabilitation goals may overlap substantially in the context of multiple sequential cancer treatments (4).

**Figure 3:**
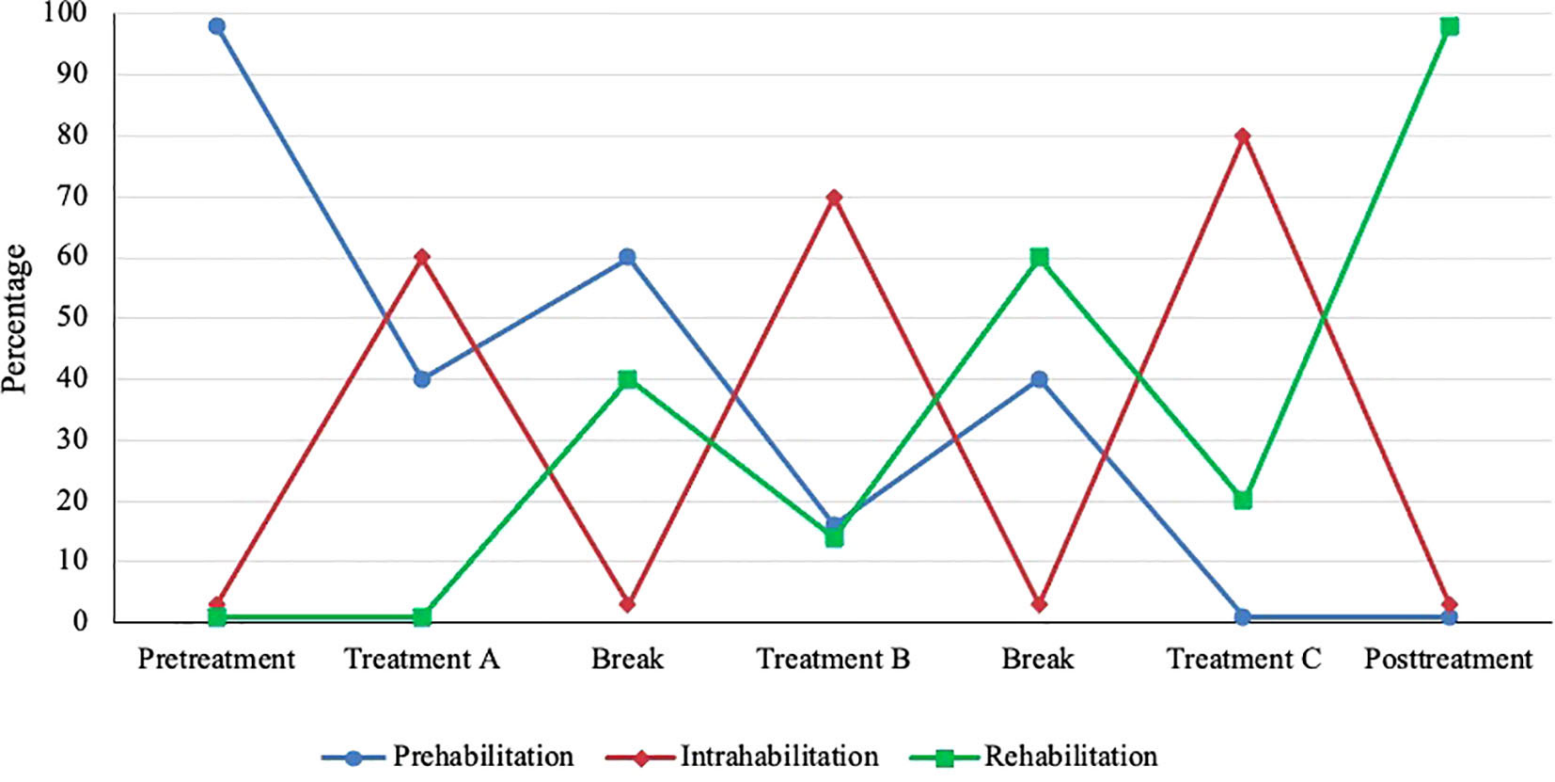
Hypothetical emphasis on prehabilitation, intrahabilitation, and rehabilitation across multiple sequential cancer treatments. Relative emphasis may vary depending on the combination and sequencing of treatments.

In classical conceptualizations of prehabilitation and rehabilitation based on acute insult/injury, rehabilitation begins when prehabilitation ends. In the EPiCC Framework, the relative emphasis on prehabilitation and rehabilitation may gradually change over the course of multiple sequential cancer treatments and may depend on the particular combination and sequencing of cancer treatments. The shift from prehabilitation to rehabilitation may happen gradually across multiple sequential treatments rather than abruptly as typically happens after surgical monotherapy. In general, prehabilitation may be more important early in the treatment sequence and diminish in importance later in the treatment sequence, whereas the opposite may be true for rehabilitation (**Figure 3**). Given the diverse roles of exercise for supportive care across multiple sequential cancer treatments, the term prehabilitation in the EPiCC Framework is restricted to the time between diagnosis and the first treatment. This phase of the postdiagnosis cancer continuum is primarily focused on prehabilitation. Similarly, the term rehabilitation in the EPiCC Framework is restricted to the time after the completion of the final treatment in a sequence. This phase of the postdiagnosis cancer continuum is primarily focused on rehabilitation.

### Intrahabilitation exercise

In the EPiCC Framework, exercise during nonsurgical cancer treatment(s) modalities is referred to as intrahabilitation (**Table 6**). Intrahabilitation may occur during a single nonsurgical treatment modality (e.g., chemotherapy) or during a concurrent combination of nonsurgical treatment modalities (e.g., chemoradiation therapy, chemoimmunotherapy). The primary goal of exercise during any (toxic) nonsurgical cancer treatment is to prevent decline and manage (tolerate) the current treatment(s). In the context of multimodal sequential cancer treatments, exercise during a cancer treatment may also include rehabilitation from any previous treatment(s) and/or prehabilitation for any subsequent treatment(s). The relative importance of prehabilitation, intrahabilitation, and rehabilitation may vary depending on the particular combination and sequencing of cancer treatment modalities (**Figure 3**). For example, during adjuvant chemotherapy after a conservative surgery and before radiation therapy, intrahabilitation during chemotherapy may be more important than rehabilitation from surgery or prehabilitation for radiation therapy. Conversely, during adjuvant radiation therapy after radical surgery and before adjuvant chemotherapy, rehabilitation from surgery and prehabilitation for chemotherapy may be more important than intrahabilitation for radiation therapy. Moreover, the relative importance of intrahabilitation may increase over the course of multiple sequential cancer treatments because of accumulating or compounding toxicities (**Figure 3**).

### Interhabilitation exercise

In the EPiCC Framework, exercising between cancer treatments is referred to as interhabilitation (**Table 6**). Although the time period between successive cancer treatments may be short, gaps of 4-6 weeks may be sufficient for a meaningful exercise intervention (60). Planned treatment breaks may occur between two different treatment modalities or even within a single treatment modality such as endocrine therapy or immunotherapy (i.e., structured treatment interruptions or “drug holidays”). By definition, exercise between treatments would serve the primary goals of both rehabilitation and prehabilitation. The relative importance of prehabilitation and rehabilitation may depend on the specific combination and sequence of treatments. For example, during the window between surgery and adjuvant chemotherapy, rehabilitation from surgery may be more important than prehabilitation for chemotherapy after radical surgery whereas the opposite may be true after conservative surgery. Moreover, the relative importance of prehabilitation and rehabilitation may also depend on whether the interhabilitation occurs early or late in the treatment sequence (**Figure 3**).

Interhabilitation exercise specifically targets the time period between two predefined treatments. For example, an exercise intervention may target the time period between neoadjuvant chemoradiation therapy and radical surgery to help patients recover from the neoadjuvant therapy and prepare for surgery (35). Conversely, an exercise intervention that targets patients after a radical surgery without regard for subsequent treatments is more likely focused on rehabilitation than interhabilitation (61). If there is no meaningful break between two sequential cancer treatments, then the supportive care role for exercise is successive intrahabilitation (e.g., intrahabilitation for chemotherapy followed by intrahabilitation for radiation therapy).

### Perihabilitation exercise

Although perihabilitation exercise is not its own discrete cancer treatment-related time period, it may be used to describe exercise interventions that occur across or around multiple contiguous cancer treatment-related time periods (e.g., before, during, between, and/or after treatments) (53) (**Tables 6**, **7**). Exercise oncology researchers may study perihabilitation exercise for a specific treatment (e.g., surgery, stem cell transplant, chemotherapy) or even a combination of treatments (e.g., surgery followed by chemotherapy). Perihabilitation goals may include combinations of prehabilitation, intrahabilitation, and/or rehabilitation. The emergence of the modern cancer treatment landscape makes perihabilitation research in exercise oncology increasingly necessary and important.

**Table 7:** Hypothetical treatment sequence of surgical resection followed by chemotherapy and radiation therapy.

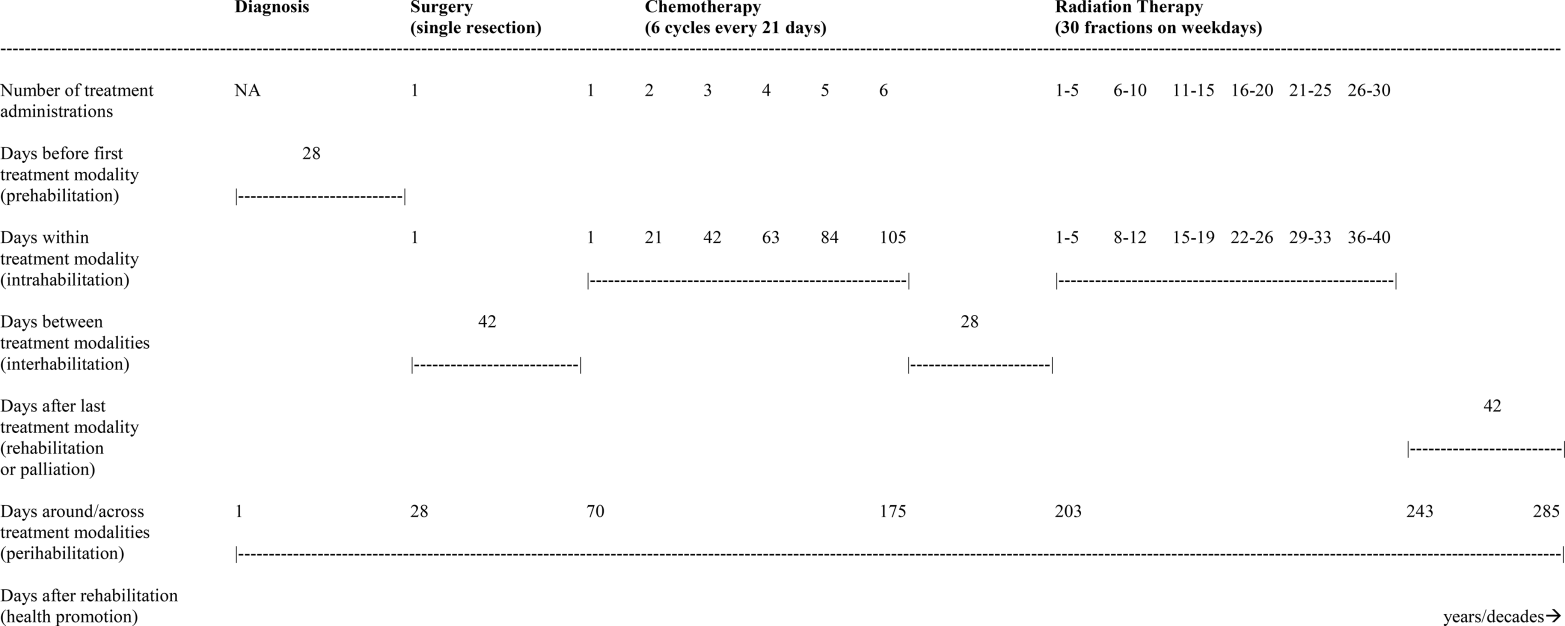

For example, Morielli et al. (26) examined the effects of exercise in rectal cancer patients during and after neoadjuvant chemoradiation therapy prior to surgery compared to usual care (i.e., perihabilitation for neoadjuvant chemoradiation therapy). This study examined intrahabilitation exercise during neoadjuvant chemoradiation therapy and interhabilitation exercise between neoadjuvant chemoradiation therapy and surgery to help patients manage the neoadjuvant chemoradiation therapy, recover from the neoadjuvant chemoradiation therapy, and prepare for surgery. Similarly, Scott et al. (62) examined the effects of exercise in breast cancer patients during chemotherapy, after chemotherapy, or both during and after chemotherapy compared to usual care. This study, therefore, compared whether perihabilitation exercise for chemotherapy (during and after) was superior to intrahabilitation exercise alone or rehabilitation exercise alone in helping breast cancer patients manage (tolerate) and recover from chemotherapy.

### Health promotion/disease prevention exercise

In the EPiCC Framework, exercise for health promotion/disease prevention begins after rehabilitation from successful cancer treatments and continues during survivorship until or unless a recurrence or second cancer occurs. Exercise during this phase focuses on improving overall health and preventing secondary diseases/late effects. The primary goals of exercise during cancer survivorship, therefore, are to improve general health outcomes such as quality of life, psychological well-being, and energy levels, as well as prevent secondary diseases/late effects. This distinct supportive care role for exercise is important because many cancer patients are cured of their cancer, experience minimal long-term complications, and return to normal life.

The goal of preventing secondary diseases/late effects is important during survivorship because some cancer patients are at higher risk of secondary diseases such as cardiovascular disease, diabetes, and osteoporosis because of their cancer and treatments. Moreover, many early stage cancer patients are more likely to die from other secondary diseases than cancer. Consequently, disease prevention may be a critical focus during the survivorship phase depending on the combination and sequencing of previous treatments. Although the prevention of secondary diseases/late effects is the primary concern of the survivorship phase, it may also be a focus earlier in the treatment trajectory for treatment(s) that are known to increase the risk of secondary diseases (e.g., some hormone therapies and chemotherapies).

For cancer survivors with lingering or chronic health effects after successful treatments, exercise during survivorship may consist of a combination of rehabilitation and health promotion/disease prevention goals. The relative emphasis on rehabilitation versus health promotion/disease prevention after successful cancer treatments may depend on the cancer treatment combination. In general, rehabilitation exercise may be more important early after treatment and diminish further out from treatment whereas the opposite may be true for health promotion/disease prevention exercise. For cancer survivors with persistent or permanent side effects, rehabilitation goals may remain important throughout survivorship and the transition from rehabilitation to health promotion/disease prevention may never occur completely.

Exercise for health promotion/disease prevention may also be the primary focus of the unique setting of active surveillance where newly diagnosed cancer patients do not immediately receive any treatments. These patients essentially enter the “survivorship” phase at the time of diagnosis and, therefore, the supportive care role of exercise in this setting may focus immediately on health promotion and disease prevention. Nevertheless, many of these patients will experience cancer progression and will ultimately require cancer treatments (44). For these patients, the supportive care role of exercise may also include prehabilitation. Given that it is unknown ahead of time which patients might progress, exercise for supportive care during active surveillance may include both prehabilitation and health promotion/disease prevention goals. Once the decision is made to undergo cancer treatment, exercise goals may shift entirely to prehabilitation.

### Palliative exercise

In the EPiCC Framework, exercise for palliation begins after stopping all life-prolonging cancer treatments for progressive disease and continues until death. Palliation is generally defined as relieving symptoms, suffering, and improving quality of life without curing the underlying terminal or life-limiting disease (63). The primary goal of exercise for palliation at the end of life is to relieve symptoms and suffering, and to maintain physical functioning and quality of life for as long as possible. This distinct supportive care role for exercise is important because almost half of all cancer patients ultimately die from the disease and are likely in need of palliation. Exercise for palliation may be anticipated as patients with advanced cancer undergo their final life-prolonging treatments. Therefore, exercise during the final treatments may consist of a combination of intrahabilitation and palliation goals. The relative importance of intrahabilitation and palliation in patients with advanced cancer may shift over successive lines of therapy and depend on the particular treatment combinations. Once the decision is made to stop cancer treatments altogether, exercise goals may shift entirely to palliation.

## Discussion

The EPiCC Framework provides structure to the field of exercise oncology and highlights the potential roles of exercise after a cancer diagnosis. In the following sections, we provide a brief discussion of future research directions although there are many more that we do not discuss. Moreover, we note some of the limitations of the EPiCC framework and how it might be applied in future systematic reviews and meta-analyses. Finally, we provide a conclusion and succinct summary of the key propositions of the EPiCC Framework.

### Emerging research questions and future directions

The EPiCC Framework highlights many new research questions and future directions for the field of exercise oncology that emerge in the context of multimodal sequential cancer treatments. **Table 8** highlights some of these key questions. Answers to these questions will help exercise oncology researchers determine the optimal positioning of exercise within different cancer treatment combinations and sequences. From a treatment perspective, (a) preclinical *in vitro* studies have studied exercise as an adjuvant monotherapy (64), (b) preclinical animal studies have studied exercise as a monotherapy (14) or concurrent therapy (24) for primary tumors (i.e., primary, neoadjuvant, or induction therapy) and/or disseminated tumor cells (i.e., adjuvant therapy), (c) observational studies have primarily examined exercise as a maintenance monotherapy (6), and (d) intervention studies have primarily studied exercise as a concurrent neoadjuvant therapy although isolated studies have been conducted as concurrent adjuvant therapy, primary monotherapy, and maintenance monotherapy (24).

**Table 8:** Exercise oncology research questions in the context of the modern cancer treatment landscape.

**General Questions**
When is the best time to exercise during multimodal sequential cancer treatments? Does the best time to exercise depend on the combination and sequence of cancer treatments? Do exercise effects early in the treatment sequence alter exercise effects later in the treatment sequence? Should the exercise prescription be modified across multimodal sequential cancer treatments? Does the optimal exercise prescription depend on the combination and sequence of cancer treatments?
Disease Treatment Questions
What is the most likely cancer treatment role for exercise as a monotherapy or concurrent therapy (e.g., priming, primary, neoadjuvant, induction, bridging, adjuvant, consolidation, maintenance, or salvage)? What is the optimal positioning of exercise for treatment effects? Does the optimal positioning of exercise for treatment effects depend on the combination and sequence of other cancer treatments? What is the optimal exercise prescription for treatment effects? Does the optimal exercise prescription for treatment effects depend on the combination and sequence of other cancer treatments?
Supportive Care Questions
What does exercise prehabilitation mean in the context of multimodal sequential cancer treatments? What does exercise rehabilitation mean in the context of multimodal sequential cancer treatments? Should exercise prescriptions during treatments focus on intrahabilitation for the current treatment, rehabilitation from the previous treatment, or prehabilitation for the subsequent treatment? Should exercise prescriptions during interhabilitation focus on rehabilitation or prehabilitation? Does the relative importance of exercise for prehabilitation, intrahabilitation, and rehabilitation depend on the combination and sequence of cancer treatments?

Studying exercise as a cancer treatment across the modern cancer treatment landscape may be challenging for preclinical studies. It may be feasible to examine exercise as a combination therapy in animal models in relation to a single cancer treatment (or a concurrent combination of cancer treatments), however, it may be more difficult to examine exercise in relation to multiple sequential treatments. That is, exercise may be tested before, during, and/or after a single (or concurrently combined) nonsurgical therapy as has been demonstrated in preclinical drug studies (65–67) but perhaps not across multiple sequential cancer treatments. Another possible approach to studying exercise after cancer treatments in preclinical models may be to use patient derived xenografts that have been previously treated. Identifying the mechanisms of action for any exercise effects will be a critical component of research integrating exercise into existing cancer treatment combinations.

Observational studies of exercise as a cancer treatment need to be initiated at diagnosis and tie assessments to the treatment-related time periods (6). Observational studies are well-positioned to study the specific role of exercise as a cancer treatment in single discrete treatment-related time periods or across multiple contiguous or disconnected treatment-related time periods to determine the best combination and sequencing of exercise within different cancer treatment combinations (6). Such studies may also become a model for how to integrate exercise assessments into routine clinical practice at critical phases of the cancer treatment experience. Intervention studies should be able to study the effects of exercise in single discrete treatment-related time periods or across multiple consecutive treatment-related time periods that are close together.

The supportive care role of exercise in cancer has been well-established across many different patient groups and treatment modalities. Nevertheless, most supportive care research has examined exercise within a single discrete treatment-related period such as before surgery, during nonsurgical therapy (especially chemotherapy and radiation therapy), long-term after curative treatments, or in patients with metastatic cancer. That is, exercise has been primarily studied as prehabilitation for surgery (68), intrahabilitation for chemotherapy (69) and radiation therapy (70), health promotion for long-term cancer survivors (71), and palliation for patients with advanced cancer (72). Some studies have examined exercise interhabilitation between neoadjuvant therapy and surgery (35).

Studies are needed to target other single discrete treatment-related periods (e.g., before, during, between, or after a specific treatment) and multiple contiguous or disconnected treatment-related periods (e.g., before, during, and between two or more sequential treatments). For example, few studies have examined exercise during other single discrete treatment-related periods such as prehabilitation for nonsurgical therapies, intrahabilitation for newer nonsurgical therapies (e.g., immunotherapy, targeted therapy), intrahabilitation for combined concurrent therapies (e.g., chemoradiation therapy, chemoimmunotherapy), or interhabilitation between sequential treatments. Moreover, when examining exercise for intrahabilitation, few studies have distinguished between the particular sequence of a therapy (e.g., chemotherapy as primary, neoadjuvant, induction, adjuvant, consolidation, maintenance, or salvage therapy) or the particular sequencing combination (e.g., adjuvant chemotherapy after radical surgery versus after primary radiation therapy). Finally, few studies have examined exercise across multiple contiguous or disconnected treatment-related periods such as before, during, between, and/or after multiple sequential treatments (i.e., perihabilitation) (4).

Perihabilitation studies in exercise oncology may be particularly informative because they evaluate the relative effects of performing exercise during single versus multiple contiguous cancer treatment-related time periods (i.e., simple versus “compound” research questions). For example, a study comparing exercise during and after chemotherapy (perihabilitation) to exercise after chemotherapy (rehabilitation) can shed light on which benefits of exercise during chemotherapy (intrahabilitation) can be recouped quickly, slowly, or not at all with rehabilitation exercise (73). The relative importance of exercise during chemotherapy depends not just on its demonstrated benefits but also on how quickly (and completely) those benefits can be recouped with exercise after chemotherapy. Similarly, a study comparing exercise before and after surgery (perihabilitation for surgery) to exercise after surgery (rehabilitation) can shed light on which benefits of exercise before surgery (prehabilitation) can be recouped quickly, slowly, or not at all with rehabilitation exercise.

In general, the case for exercise early in the cancer treatment sequence is strengthened if there are benefits that cannot be recouped completely or at all by exercise later in the cancer treatment sequence. Such information may allow exercise oncology practitioners to reduce or “de-escalate” the exercise prescription at certain times during a particular cancer treatment combination rather than promote the “maximally tolerated dose” of exercise across the entire postdiagnosis cancer continuum. Finally, from a treatment perspective, exercise effects earlier in the treatment sequence may alter exercise effects later in the treatment sequence (i.e., “compound” research questions). It is unclear if continuous exercise from the time of diagnosis and for the balance of life is optimal from a treatment perspective or whether there are critical time points during a treatment sequence when exercise may exert optimal treatment effects (i.e., exercise breaks with rechallenge).

Future studies are also needed that examine the effects of different exercise prescriptions on supportive care and disease outcomes including the type, frequency, intensity, duration, progression, and periodization. Moreover, it will be important to examine the specific timing of individual exercise administrations in relation to different cancer treatment schedules. That is, it will be important to specify how to integrate the treatment schedule of exercise with the treatment schedule of the cancer therapy.

Finally, the modern cancer treatment landscape also has significant implications for the feasibility and sustainability of exercise across multiple sequential cancer treatments. Exercise motivation and behavior change is difficult during a single cancer treatment and may become even more challenging in the context of the modern cancer treatment landscape. Conceivably, the determinants of exercise may vary depending on the combination and sequencing of cancer treatments. For example, the determinants of exercise during neoadjuvant chemotherapy may be different than during adjuvant chemotherapy. Similarly, the determinants of exercise during radiation therapy after surgery may be different than during radiation therapy after chemotherapy. Exercise behavior change interventions may need to take into account the particular combination and sequencing of cancer treatments.

### Limitations

There are important limitations of the EPiCC Framework and our overview of the field of exercise oncology. First, our proposed framework does not reflect all of the clinical scenarios in oncology, although we believe it covers the major current treatment paradigms. Moreover, we believe our framework may be extrapolated to other less common treatment combinations or to new treatment combinations as they emerge in the future. Second, the roles of exercise as a disease treatment and supportive care intervention will vary by the specific types of cancer treatments within the cancer treatment combinations (e.g., type of surgery, type of chemotherapy, type of immunotherapy). Third, the roles of exercise as a disease treatment and supportive care intervention will also vary by the type and subtype of cancer (e.g., pancreatic, liver, brain, diffuse large B-cell lymphoma) and not just by the cancer treatment combinations. This precision medicine approach to exercise oncology is critical as the response to exercise may be quite different for different cancers, subtypes, cell lines, molecular profiles, and mutational statuses. Therefore, the EPiCC Framework will need to be applied and tested for specific cancers and subtypes. Fourth, it is unclear how feasible it will be to conduct preclinical *in vitro*, preclinical animal, observational, and clinical studies within and across multiple sequential cancer treatments. Innovative ideas will be needed to overcome logistical and methodological challenges in future research. Finally, we did not conduct systematic reviews on the effects of exercise within each of the different cancer treatment-related time periods and cancer treatment combinations. Such an undertaking was deemed impractical and beyond the scope of this paper. Future systematic reviews may consider using the EPiCC Framework to target their reviews to a specific treatment-related time period or across multiple contiguous treatment-related time periods within a specific cancer treatment combination. Alternatively, future systematic reviews may consider using the EPiCC Framework to organize and structure their reviews for a specific type of cancer (e.g., bladder, pancreatic, ovarian).

## Conclusions

**Figure 4:**
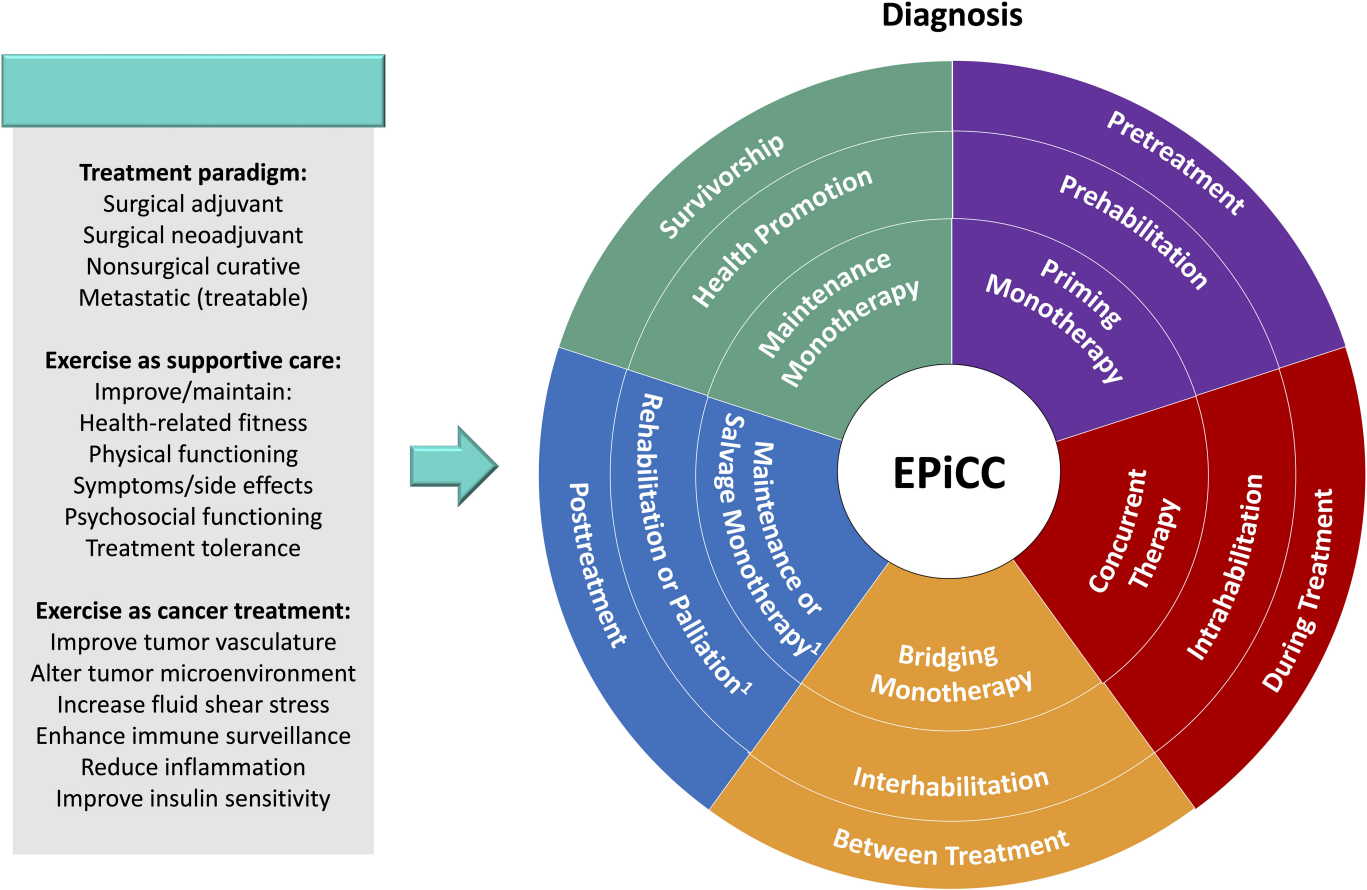
Exercise Across the Postdiagnosis Cancer Continuum (EPiCC) Wheel. Outer ring contains treatment-related time periods. Middle ring contains supportive care roles for exercise. Inner ring contains cancer treatment roles for exercise. ^1^Rehabilitation and maintenance monotherapy if successful treatment; palliation and salvage monotherapy if unsuccessful treatment.

In conclusion, the modern cancer treatment landscape has become saturated and complicated for many cancers. Exercise may play many important distinct roles across the postdiagnosis cancer continuum, however, studies need to account for the positioning of exercise in relation to the combination and sequencing of other cancer treatments. The EPiCC Framework provides a conceptual model to organize the potential roles of exercise across the postdiagnosis cancer continuum (**Figure 4**). Before cancer treatments, exercise may serve as prehabilitation for any cancer therapy and as a possible priming monotherapy for any nonsurgical therapy. During cancer treatments, exercise may serve as intrahabilitation and as a concurrent therapy for any nonsurgical therapy at any point in the treatment sequence (e.g., primary, neoadjuvant, induction, adjuvant, consolidation, maintenance, or salvage). Between cancer treatments, exercise may serve as interhabilitation and as a bridging monotherapy between any two treatments in the treatment sequence. Immediately after successful treatments, exercise may serve as rehabilitation and as a maintenance monotherapy. During survivorship, exercise may be implemented for health promotion/disease prevention as well as a maintenance monotherapy. During end of life, exercise may serve as palliation but it is unlikely to serve as a salvage monotherapy. We propose the EPiCC Framework to stimulate a more targeted, integrated, and clinically-informed approach to the study of exercise after a cancer diagnosis.

## Data Availability

The original contributions presented in the study are included in the article/supplementary material. Further inquiries can be directed to the corresponding author.
